# Pimozide and Imipramine Blue Exploit Mitochondrial Vulnerabilities and Reactive Oxygen Species to Cooperatively Target High Risk Acute Myeloid Leukemia

**DOI:** 10.3390/antiox10060956

**Published:** 2021-06-15

**Authors:** Zhengqi Wang, Tian Mi, Heath L. Bradley, Jonathan Metts, Himalee Sabnis, Wandi Zhu, Jack Arbiser, Kevin D. Bunting

**Affiliations:** 1 Division of Hem/Onc/BMT, Department of Pediatrics, Emory University, Atlanta, GA 30322, USA; zhengqi.wang@emory.edu (Z.W.); jasmine.mi@roche.com (T.M.); heath.lewis.bradley@emory.edu (H.L.B.); jmetts1@jhmi.edu (J.M.); hsabnis@emory.edu (H.S.); wzhu3@gsu.edu (W.Z.); 2 Aflac Cancer and Blood Disorders Center, Children’s Healthcare of Atlanta, School of Medicine, Emory University, Atlanta, GA 30322, USA; 3 Department of Dermatology, Emory University, Atlanta, GA 30322, USA; jarbise@emory.edu; 4 Veterans Administration Medical Center, Atlanta, GA 30322, USA

**Keywords:** acute myeloid leukemia, Flt3-internal tandem duplication, reactive oxygen species, mitoSox, BH3 mimetic, mTOR inhibitor, repurposed drugs

## Abstract

Acute myeloid leukemia (AML) is a heterogeneous disease with a high relapse rate. Cytokine receptor targeted therapies are therapeutically attractive but are subject to resistance-conferring mutations. Likewise, targeting downstream signaling pathways has been difficult. Recent success in the development of synergistic combinations has provided new hope for refractory AML patients. While generally not efficacious as monotherapy, BH3 mimetics are very effective in combination with chemotherapy agents. With this in mind, we further explored novel BH3 mimetic drug combinations and showed that pimozide cooperates with mTOR inhibitors and BH3 mimetics in AML cells. The three-drug combination was able to reach cells that were not as responsive to single or double drug combinations. In Flt3-internal tandem duplication (ITD)-positive cells, we previously showed pimozide to be highly effective when combined with imipramine blue (IB). Here, we show that Flt3-ITD^+^ cells are sensitive to an IB-induced dynamin 1-like (Drp1)-p38-ROS pathway. Pimozide contributes important calcium channel blocker activity converging with IB on mitochondrial oxidative metabolism. Overall, these data support the concept that antioxidants are a double-edged sword. Rationally designed combination therapies have significant promise for further pre-clinical development and may ultimately lead to improved responses.

## 1. Introduction

Acute myeloid leukemia (AML) is a devastating disease that has remained difficult to treat despite major advances in understanding the mechanisms of leukemogenesis. AML has classically been treated with combination chemotherapy that includes anthracyclines and cytarabine [1]. Receptor tyrosine kinase-based drug inhibition strategies brought much promise to the treatment of AML with Flt3 mutations, but resistance mechanisms have been problematic. More general approaches that induce apoptosis based on the use of BH3 mimetics [2,3] have been a very promising new development in AML therapy in recent years. Since many cancer drugs impinge on the mitochondrial intrinsic apoptosis pathway, metabolic vulnerabilities of AML have been pursued in pre-clinical and ongoing clinical studies. Prospective characterization of AML samples using BH3 profiling [4] have shown that patients vary in their responsiveness to BH3 mimetics and that some patients can be carefully chosen to be good responders, especially when used in combination with chemotherapy [5]. The evolution of BH3 mimetics has gone from first-generation experimental agents targeting Bcl-2 and Bcl-X_L_, used primarily in mouse models (ABT-737), to formulations optimized for oral administration (ABT-263, Navitoclax; highly binds Bcl-2, Bcl-X_L_, Bcl-w), to second-generation agents that are more precise in binding only Bcl-2 (ABT-199, Venetoclax) and sparing Bcl-X_L_ and consequent effects on platelet survival and thrombocytopenia. Notably, all three forms of this BH3 mimetic are equally effective in vitro on culture leukemia cells.

Major advances in the metabolic understanding of common features of AML survival mechanisms have been made in recent years. A variety of mutations are observed and underlying these signaling changes may be a common dysregulation of the metabolic state and changes in mitochondria turnover (fusion and fission) [6,7]. High Flt3 mutational load correlates with BH3 mimetic-induced apoptosis sensitivity, but gaining a greater response throughout the gradient of sensitivity has been a confounding factor. Flt3-independent expression of Bcl-2 is a major mechanism of resistance to Flt3 inhibitors that can be overcome by BH3 mimetics [8]. Mitochondrial oxidative phosphorylation in AML has an oncogenic driver-like activity and is believed to contribute to clonal evolution during relapse. Therefore, metabolism and AML have been examined closely in recent years and metabolic vulnerabilities have been suggested [9,10,11]. For example, enhanced glycolysis can contribute to reduced sensitivity to agents such as Ara-C [12]. Targeting mitochondrial oxidative metabolism has also been suggested as a means to target the Achilles’ heel of leukemia stem cells. Currently, the relationship between high risk Flt3-internal tandem duplications (ITD)^+^ AML and intermediate risk Flt3-tyrosine kinase domain (TKD) or Flt3-ITD^+^ Nucleophosmin (NPM1)^+^ AML has not been clearly established in relation to BH3 mimetic usage.

Signal transducer and activator of transcription 5 (STAT5) is persistently activated in the high/intermediate risk AML subsets expressing mutant Flt3 or Kit receptors. Our lab has previously demonstrated that STAT5 induces Bcl-2 expression which is essential to off-set the pro-apoptotic impact of other STAT5 regulated genes [13,14], which can include active p53, Cish, and Socs1. This “priming” for cell death by STAT5 is common to other oncogenes such as Ras, where oncogene-induced senescence is counterbalanced by strong pro-survival signals [15]. Recruitment of Mcl1 is one such signal that has been characterized in AML. Mcl1 can be induced directly by STAT5 or by cross-activation of the Akt/mTOR pathway [16]. STAT5 cross-talk with Akt to drive glycolysis has been reported in lymphocytes and myeloproliferative neoplasms (MPNs), but has not been directly tested in AML despite changes in Glut1 expression when STAT5 is inhibited [17,18,19]. Therefore, targeting STAT5 may impact upon drug sensitivity in these specific AML subsets and represents a candidate for developing a novel combination regimen. In our prior work, we demonstrated that imipramine blue (IB), a novel chimeric drug (combining a triphenylmethane dye with the antidepressant imipramine [20]) created by Jack Arbiser at Emory University, could target STAT5 activation at higher doses (300 nM) but was able to induce cell death at concentrations below that required for STAT5 inhibition (100−150 nM) [21]. Therefore, this study was performed to determine the phospho-STAT5-independent mechanisms of cell death induced by IB in AML.

Single agent BH3 mimetic usage leads to resistance and is increasingly recognized as a clinical problem. Since patient responses are transient with monotherapy, new approaches are needed. Combinations with 5-aza-cytidine was recently given accelerated approval by the FDA. Additional combinations with MEK and PI3K inhibitors have been reported [22,23,24,25]. Most of these phospho-STAT5-independent approaches target epigenetic regulation of cell survival mechanisms. Pimozide (Pim) also has potent cooperative function with IB [21] and has anti-psychotic functions that include calcium channel blocking activity. In this report, we demonstrate novel combinations based on the antidepressant derivative imipramine blue plus the antipsychotic drug pimozide that act through mitochondrial ROS liberation. This study improves the understanding of the mechanisms of resistance and identifies rationale combinations that exploit metabolic vulnerabilities in AML. Furthermore, it suggests that caution should be given in regard to antioxidants in leukemia therapy, an emerging area of importance for understanding tumorigenesis and developing effective targeting strategies [26].

## 2. Materials and Methods

### 2.1. Cell Culture

All cultured cell lines were originally sourced from the American Type Culture Collection (ATCC) or DSMZ (OCI-AML3 cells) prior to being obtained from other laboratories. Cells were grown in media containing 1% penicillin/streptomycin and were negative for mycoplasma contamination prior to use. The Flt3-ITD^+^ cell line MV4-11 representing homozygous Flt3-ITD mutation and MOLM-13 representing heterozygous Flt3-ITD mutation were obtained from William Tse (MetroHealth System, Cleveland, OH, USA). MV4-11 cells were grown in IMDM supplemented with 10% FBS, and MOLM-13 cells were grown in RPMI supplemented with 10% FBS. Additional Flt3-ITD^neg^ cell lines included: OCI-AML3, which was grown in αMEM supplemented with 10% FBS, and HL-60, HEL, CCRF-288, THP-1, Kasumi-1, NB4, Nomo-1, and K562 (blast crisis CML), which were gifts from Gang Huang (Ohio State University) and William Tse. HL-60 cells were grown in IMDM with 20% FBS. HEL and K562 cells were grown in RPMI +10% FBS. All AML cell lines were tested for authenticity by short-tandem repeat (STR) analysis in the Emory Integrated Genomics Core Facility.

### 2.2. Drugs

STAT5 inhibitor pimozide (P1793) was purchased from Sigma-Aldrich Inc. (St. Louis, MO, USA). Imipramine HCl, ABT-737, ABT-263, ABT-199 were purchased from Selleck Chemicals (Houston, TX, USA) and used interchangeably in these studies. Initial experiments began with ABT-737 and as additional more clinically relevant forms were available the studies moved to ABT-263 and finally to ABT-199. These BH3 mimetics have identical function in vitro on cultured AML cells. AZD 8055 was purchased from Selleck Chemicals (Houston, TX, USA). Imipramine blue and gentian violet were provided by the Jack Arbiser lab. Mibefradil (M5441) and fluoxetine (F132) were purchased from Sigma-Aldrich Inc. (St. Louis, MO, USA) as indicated. DPI was obtained from Sigma-Aldrich Inc. (St. Louis, MO, USA) (D2926).

### 2.3. Cell Survival and ROS Assays

For the cytotoxicity assay, after 48 h of drug treatments, cells were analyzed by trypan blue exclusion assay as previously described [27]. Briefly, trypan blue exclusion assay was routinely performed to assess drug sensitivity. Cells that were clear or slightly pink were scored as live cells and cells that were blue/dark blue were scored as dead/dying cells based on increased membrane permeability to the trypan blue dye. ROS was measured 4 h after the indicated treatment by flow cytometry using the CytoFLEX Flow Cytometer (Beckman Coulter, Braille, CA, USA) following staining with MitoSOX™ Red Mitochondrial Superoxide Indicator (Thermo Fisher Scientific, Waltham, MA, USA, M36008) according to the manufacturer’s instructions. NAC was purchased from Sigma-Aldrich Inc. (St. Louis, MO, USA) (A9165). All flow cytometry was performed using the Emory Winship/Children’s Pediatric Flow Cytometry core. The data analysis was performed using FlowJo software (BD).

### 2.4. Real-Time Quantitative Reverse Transcriptase-Polymerase Chain Reaction (qRT-PCR) and Caspase-3 Analyses

Total cellular RNA was extracted from 1 × 10^6^ cells using Trizol reagent (Invitrogen, Waltham, MA, USA) according to the manufacturer’s protocol. cDNA was synthesized from 1.0 μg of RNA using a SuperScript III First Strand Synthesis System (Invitrogen, Waltham, MA, USA). qRT-PCR was performed using an iQ SYBR Green Supermix (Bio-Rad) and amplification was performed on a 7500 Real-time PCR instrument (Applied Biosystems). MV4-11 cells were treated with ABT-263 (100 nM) and analyzed 24 h later for caspase 3 using an antibody to total caspase 3. Caspase 3 cleavage was detected by a different pH species migrating in the micro-capillary electrophoresis system. Antibodies used to total caspase 3 were obtained from Cell Signaling (Danvers, MA, USA) (9662s).

### 2.5. Western Blotting

Cells were lysed in RIPA buffer with protease and phosphatase inhibitors (Roche, #04693124001 and #04906845001) for 30 min on ice followed by centrifugation for 10 min at 10,000× *g*. Protein concentrations were determined using the Bio-Rad protein assay (Bio-Rad, Heracles, CA, USA #500-0006) and proteins were separated on an SDS-polyacrylamide gel followed by transfer to either a nitrocellulose (Fisher, Waltham, MA, USA #1215471) or PVDF (Immobilon, Darmstadt, Germany, #IPVH00010) membrane. After blocking in 5% BSA for 1 h, membranes were incubated in the appropriate antibody overnight and detection was by chemiluminescence (Thermo Fisher Scientific, Waltham, MA, USA, #34080) or by the Odyssey Clx imaging system (LI-COR Biosciences, Lincoln, NE, USA). Image Studio v4.0 software was used for densitometry quantification. Antibodies for Western blot were obtained from the following: Anti-STAT5 (Phospho-STAT5 Y694) antibody (Abcam, Cambridge, UK), Phospho-Akt (Ser473) (D9E), Phospho-Akt (Thr308) (D25E6) (Cell Signaling), Phospho-DRP1 (Ser637) (D3A4) (Cell Signaling, Danvers, MA, USA), DRP1 Antibody (Novus Biologicals), p38alpha MAPK antibody and Phospho-p38alpha MAPK (Cell Signaling, Danvers, MA, USA). Primary antibody concentrations were used according to the manufacturers’ suggestions. The secondary antibody combinations used were either IRDye 680RD (925-68070)/IRDye 800CW (925-32210) or IRDye 680RD (925-68071)/IRDye 800CW (925-32211). These goat anti-rabbit (LI-COR, Lincoln, NE, USA) antibodies were used at 1:10,000 to 1:20,000 dilution.

### 2.6. Statistical and Synergy Analyses

All data were derived as a result of three or more independent experiments, unless stated otherwise. Student’s two tailed *t*-test was used to calculate *p*-values and values less than 0.05 were considered to be significant. Throughout all figures, the following nomenclature was used to indicate the level of statistical significance: * *p* < 0.05; ** *p* < 0.01; *** *p* < 0.001. Synergy was tested using the SynergyFinder web-based tool using the ZIP method and the following parameters: readout: inhibition; baseline correction: yes. (https://synergyfinder.fimm.fi, accessed on 24 May 2021). The tool tests the significance of combined drug interactions and requires dose–response data with three data points to calculate a synergy score. For ABT–AZD combinations, only one concentration (250 nM AZD) was used experimentally. In order to be able to calculate synergy, intermediate concentrations were modeled with an assumption that cell death remained at the untreated level. Using this parameter, an estimated minimum SynergyFinder score was calculated. If experimental cell death increased above baseline on any intermediate drug concentration, then synergy would increase from that calculated. This method assumes that intermediate concentrations of drug do not have opposite effects from higher concentrations.

## 3. Results

### 3.1. Pimozide Synergizes Specifically with BH3 Mimetics but Not with Other Antagonists of ROS Generation

**Figure 1:**
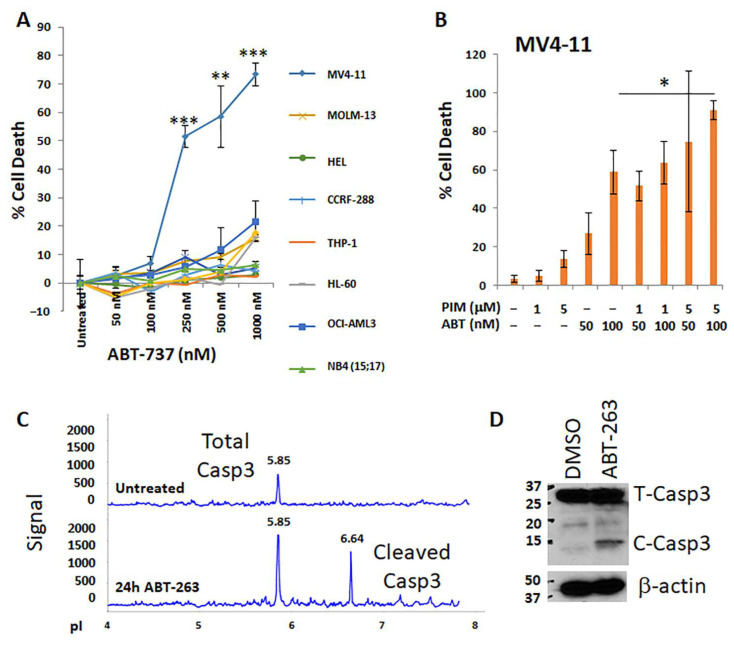
Most human AML cell lines are highly resistant to BH3 mimetic treatment except homozygous Flt3-ITD+ MV4-11 cells. (**A**) A panel of AML cell lines representing various cytogenetic and mutation status was treated by the BH3 mimetic ABT-737. The trypan blue exclusion assay was performed 48 h post-treatment. Cell death was normalized to untreated cells and in some instances survival was greater than control leading to slightly negative *y*-axis values. (*n* = 3; *p* values were calculated relative to the untreated control). * *p* < 0.05; ** *p* < 0.01; *** *p* < 0.001. (**B**) MV4-11 cells were treated for 48 h with BH3 mimetics alone and cytotoxicity was measured alone or when combined with pimozide (Pim). *p* values were calculated for MV4-11 cells with the comparisons shown (*n* = 3). (**C**) NanoPro analysis of caspase-3 for MV4-11 cells responding to ABT-263 treatment. Total and cleaved caspase 3 proteins are shown migrating at different isoelectric points (pI). *p* values are relative to the ABT 100 nM group. (**D**) Western blot analysis of caspase-3 in ABT-263 treated MV4-11 cells. Since caspase cleavage occurs on an earlier time course than survival endpoints which we always measured at 48 h), cells were stimulated with 100 nM ABT-263 for 24 h at 3 × 10^5^ cells/mL and 30 μg of protein was loaded per lane.

While pimozide has been described as a STAT 3/5 inhibitor in CML [28] and AML [29] cells, the full extent of its mechanism of action in AML has not been explored. Since we previously reported that pimozide is highly synergistic with IB-mediated calcium modulation, we wanted to explore pimozide in combination with other inducers of mitochondrial cell death. Pimozide did not synergize with rotenone, metformin, or DPI (Figure S1), thus pointing away from hydrogen peroxide as an endpoint mediator of pimozide action. We wanted to examine if pimozide alone or in combination with ABT-263 on MV4-11 cells could synergize. Although pimozide has been described as a STAT5 inhibitor, it is more generally characterized as a calcium-modulating anti-psychotic agent which is FDA-approved for patients with psychosis and/or depression. A panel of AML cell lines were screened initially for sensitivity to BH3 mimetic (ABT-737) to assess induction of cell death. To determine the intrinsic responsiveness of AML cell lines, the first generation BH3 mimetic that inhibits both Bcl-2 and Bcl-X_L_, was used to treat a panel of lines at nM concentrations. MV4-11 cells showed the most sensitivity in the low nM range as compared with a range of other sensitive cell lines of varying FAB and cytogenetic composition (Figure 1A). In MV4-11 cells, the combination of ABT-263 and pimozide was also very effective at inducing cytotoxicity (Figure 1B) with a SynergyFinder score of 34.35. Separate capillary nanoimmunoassay (NanoPro) analysis [30] of BH3 mimetic (ABT-263) treated MV4-11 cells showed increased cleavage of caspase 3 (Figure 1C). To determine apoptosis by cleavage of caspase-3, MV4-11 cells were analyzed by Western blot (Figure 1D). Associated with BH3 mimetic (ABT-263) treatment of MV4-11 cells was a corresponding increase in cleaved caspase 3. Therefore, MV4-11 cells are uniquely highly sensitive to BH3 mimetic drugs, and of all the cell lines examined, these are homozygous Flt3-ITD^+^.

### 3.2. Inhibition of mTOR Synergizes with BH3 Mimetics in Killing Flt3-ITD^+^ AML Cells

**Figure 2:**
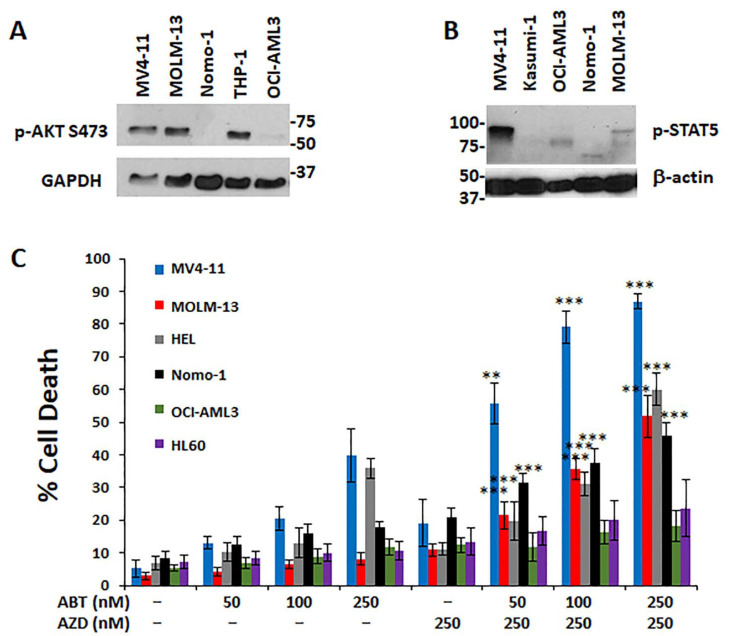
STAT5 and Akt activation are associated with BH3 mimetic resistance and downstream survival signals can be pharmacologically targeted to achieve synergistic response in Flt3-ITD^+^ cells. (**A**) pAKT expression levels was measured by Western blot analysis in several cell lines, including mainly the MV4-11 and MOLM-13 lines. (**B**) pSTAT5 expression was analyzed in MV4-11 cells and directly compared with MOLM-13 cells. (**C**) Dual drug treatments for 48 h using ABT-263 + AZD 8055 was performed to test whether they would induce greater cytotoxicity in Flt3-ITD^+^ cell lines (MV4-11 and MOLM-13). In comparison, a variety of other Flt3-ITD wild-type cell lines were also tested. (*n* = 6, *p* values were calculated relative to the AZD 250 (nM) group). ** *p* < 0.01; *** *p* < 0.001.

The effects of combination treatment with both BH3 mimetic and mTOR inhibitor were next assessed, since rapalog mTOR inhibitors have already progressed into the clinic and experimental mTORC1/2 inhibitors are available. The combination of BH3 mimetic (ABT-263) and mTOR inhibitor (AZD 8055) was synergistic in MV4-11 and MOLM-13 cells but not in the control Kasumi cells which lack Flt3-ITD expression (Figure S2). Since ABT-263 and ABT-199 (as well as ABT-737) were interchangeable for in vitro studies, the subsequent figures involve only ABT-263. To determine whether pimozide/mTOR combination is a potential signaling target in high-risk AML cells, MV4-11 and MOLM-13 cells were examined by Western blot. MV4-11 cells highly expressed both pAKT^S473^ and pSTAT5 and MOLM-13 cells also expressed high levels of pAKT^S473^ but had intermediate/low levels of pSTAT5 (Figure 2A,B). Since both AZD 8055 or pimozide inhibition showed activity on Flt3-ITD^+^ AML cells but not Kasumi1 cells, additional combinations of these agents were tested on a wider range of AML cell lines to examine specificity. As expected, mTOR inhibitor (AZD 8055) plus BH3 mimetic (ABT-263) was most selective for killing MV4-11 and MOLM-13 cells, but had more moderate selectivity for HEL and Nomo-1 (Figure 2C). In contrast, OCI-AML3 and HL-60 cells were relatively resistant to the combination treatment. The estimated minimum SynergyFinder score for the responsive cell lines was as follows: MV4-11 (19.52); MOLM-13 (6.85); HEL (5.21); Nomo-1 (4.88).

### 3.3. BH3 Mimetics Combined with Pimozide and mTOR Inhibitors Improves Sensitivity of the Most Resistant AML Cells

**Figure 3:**
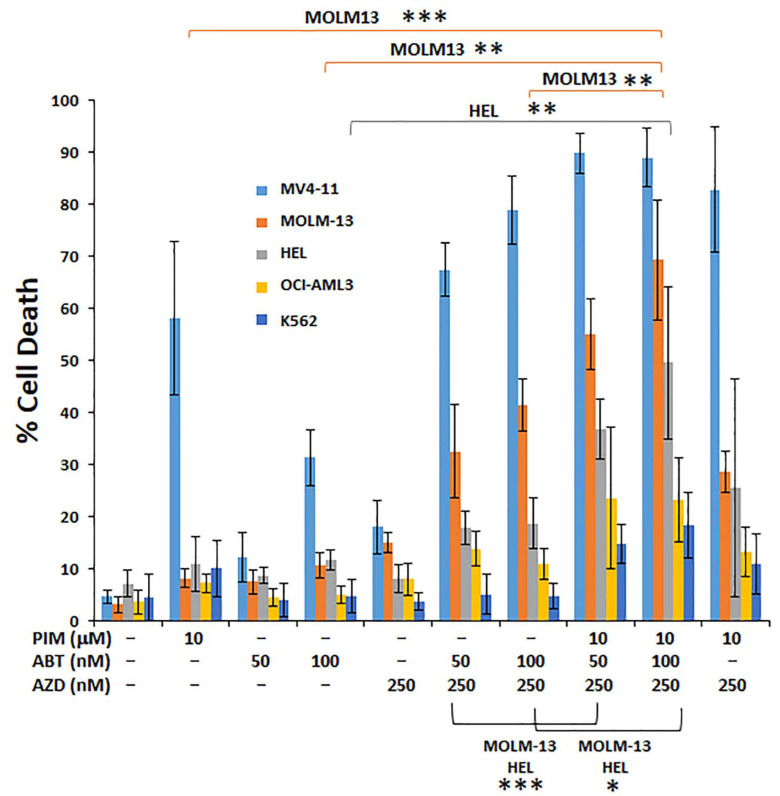
Triple combination treatment with ABT-263, AZD 8055, and pimozide selectively targets non-responders to single and dual combinations. Triple combination drug therapy after 48 h treatment is shown for cells treated with pimozide (PIM), ABT-263, and AZD 8055 (AZD). This combination was assessed in MOLM-13 and HEL cells to determine whether pimozide could effectively increase cell death determined by trypan blue exclusion assay compared with the ABT-AZD combination. *n* = 6 for all cell lines shown; *p* values were generated by comparing 2-drug vs. 3-drug combos as indicated. * *p* < 0.05; ** *p* < 0.01; *** *p* < 0.001.

Given that the properties of high-risk AML differ from lower risk subsets, there is still a need to develop agents that target a wider range of subsets. Triple drug combinations are being developed and have been found to be more efficacious in some instances. Therefore, we tested BH3 mimetic (ABT-263), mTOR inhibitor (AZD 8055), and pimozide alone or in combination on several AML cell lines (Figure 3). Pimozide alone was moderately effective on MV4-11 cells in the micromolar range. There was no significant advantage for pimozide combined with AZD 8055—compared with pimozide alone. However, the three-drug combination was observed to work best in the MOLM-13 and HEL cells, which were the weaker responders to the two-drug (ABT/AZD) combination. This experiment highlights the potential addition of pimozide to augment BH3 mimetic-mTOR inhibitor sensitivity of poorer responding AMLs, which results in significantly higher cell death in MOLM-13 and HEL cells. Altogether, these results highlight the advantage of two-drug and three-drug combinations in targeting AML cells.

### 3.4. Imipramine Blue Moieties Define Sensitivity and Specificity for Apoptosis Induction Independent of the Unfolded Protein Response

**Figure 4:**
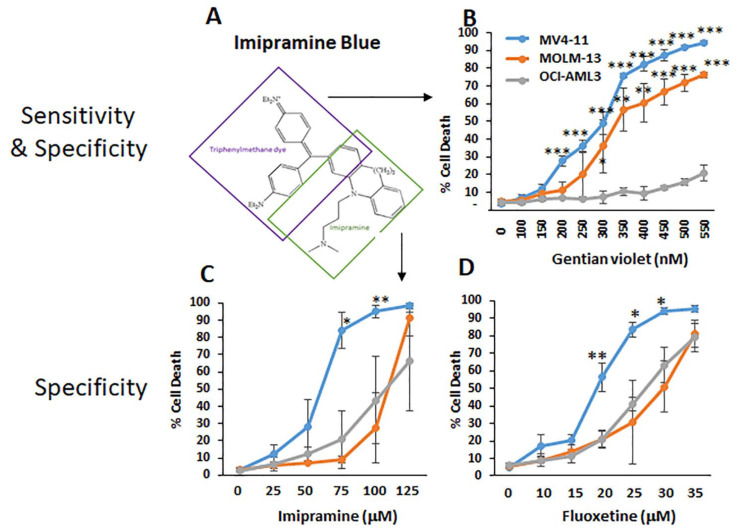
Structure–function analysis of imipramine blue reveals two moieties with AML targeting activity. (**A**) Imipramine blue is the chimeric compound generated through fusion of a triphenylmethane dye with imipramine. To determine the functional roles of the parental constituents, cytotoxicity assays were performed. (**B**) The gentian violet moiety was tested for both sensitivity and specificity for Flt3-ITD^+^ AML cells (*n* = 3). (**C**) In comparison, the imipramine moiety was also tested in MV4-11 cells (*n* = 3). (**D**) As with imipramine, another anti-depressant drug fluoxetine was tested to determine whether it would have a similar cytotoxicity profile. Note that the legend and colors for the lines are the same for panels B-D but is only included in panel D because of space constraints. *p* values for panels B-D are calculated at individual drug concentrations relative to OCI-AML3 cells at the same concentration. * *p* < 0.05; ** *p* < 0.01; *** *p* < 0.001.

We previously showed that imipramine blue (IB) was able to sensitively and selectively induce apoptosis in Flt3-ITD^+^ AML cells [21]. To examine the structure–function relationship of IB (Figure 4A), we tested the two moieties that comprise IB separately. The gentian violet moiety was the most effective on AML cells (Figure 4B), retaining both sensitivity to low nM concentrations and specificity for MV4-11 and MOLM-13 cells compared with OCI-AML3 cells. However, the imipramine moiety was also specific for MV4-11 cells but not very sensitive since μM concentrations were required (Figure 4C). In comparison, fluoxetine, another type of antidepressant drug showed a virtually identical profile to that of imipramine (Figure 4D) indicating that the common calcium release associated with antidepressants is of potentially therapeutic value in AML. Overall, these initial studies indicated that a common endpoint may be critical for pushing the IC_50_ from the μM to the nM range.

**Figure 5:**
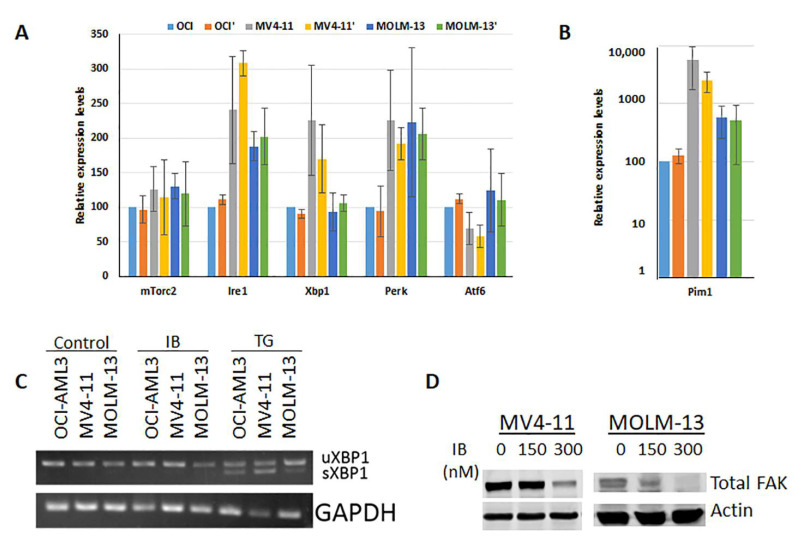
IB does not induce an unfolded protein response but does suppress expression of focal adhesion kinase. (**A**) A panel of genes related to the unfolded protein response were assayed by real-time qRT-PCR. The panel included mTorc2, Ire1, Xbp1, Perk, and Atf6. (**B**) The positive control for qRT-PCR is shown on a log scale *y*-axis since the Pim1 levels were comparably very high. *n* = 4 for all cell lines tested. *p* values were not significant (>0.05) for comparisons between IB treated and untreated samples. However, not indicated on the graph: Ire1 and Pim1 were significantly different (*p* < 0.05) relative to OCI-AML3 cells under all conditions; Xbp1 was only significant for untreated MV4-11 cells; Perk was only significant for treated MV4-11 and treated MOLM-13 cells; Atf6 was only significant for untreated MV4-11 cells. (**C**) In addition to testing gene expression changes typically present in UPR, cleavage of Xbp1 was also examined following IB treatment. Thapsigargin TG was the positive control. (**D**) In order to test for a general cell death mechanism, total FAK levels were also assessed following 24 h IB treatment in both MV4-11 and MOLM-13 Flt3-ITD^+^ cells.

Since IB induces intracellular calcium release as part of the apoptotic mechanism in Flt3-ITD^+^ AML cells, we explored the mechanism of action further. A range of genes known to be expressed during the unfolded protein response (UPR) were examined following vehicle or IB treatment (Figure 5A,B). Although the baseline levels of several genes were elevated in Flt3-ITD^+^ vs. Flt3-WT cells, IB did not induce significant changes. Likewise, examination of unspliced and spliced forms of XBP1 showed that IB was not able to induce XBP1 splicing compared with the positive control, thapsigargin (TG) (Figure 5C). Finally, consistent with an apoptotic cell death mechanism, total focal adhesion kinase (FAK) levels declined precipitously following treatment with IB at nM doses (Figure 5D). Altogether, these data indicate that the mechanism of action of IB-induced apoptosis on Flt3-ITD^+^ AML cells does not involve induction of the UPR.

### 3.5. Imipramine Blue Induces Phosphorylation and Activation of p-Drp1/p-p38 MAPK/ROS

**Figure 6:**
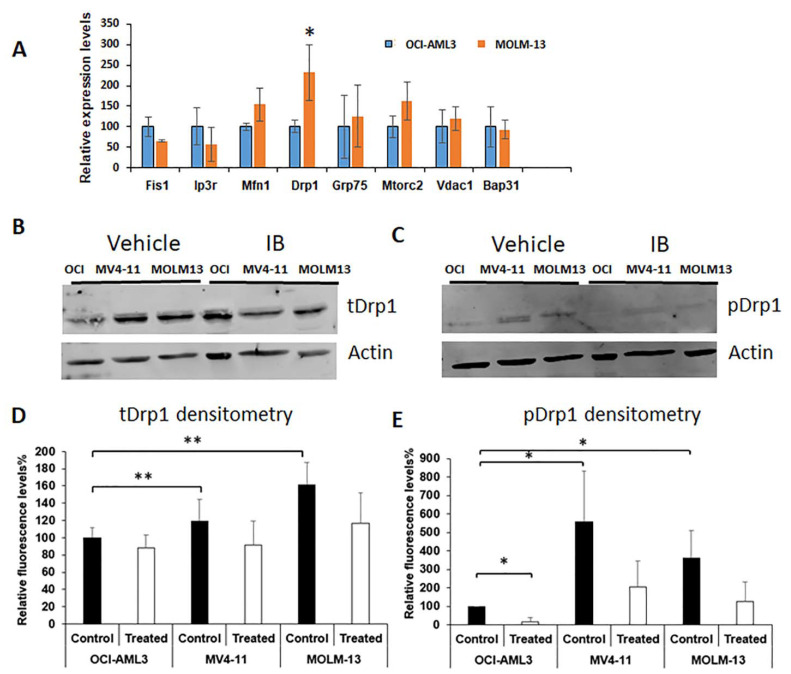
IB suppresses phosphorylation of DRP1 in Flt3-ITD^+^ cells. (**A**) A panel of genes related to the ER–mitochondria interface were assayed by real-time qRT-PCR. The panel included Fis1, Ip3r, Mfn1, Drp1, Grp75, Mtorc2, Vdac1, and Bap31 (*n* = 3 for all except Drp1 where *n* = 7). *p* values were calculated relative to OCI-AML3 cells. (**B**) Western blot analysis for total Drp1 protein with β-actin as the loading control. (**C**) Western blot analysis for phospho-Drp1 protein with β-actin as the loading control. (**D**,**E**) Densitometry analysis was performed and quantitation is shown for replicates of the Western blots in panels C and D. *n* = 3 and *p* values were calculated with comparisons as shown between either OCI-AML3 cells or with or without treatment. * *p* < 0.05; ** *p* < 0.01.

**Figure 7:**
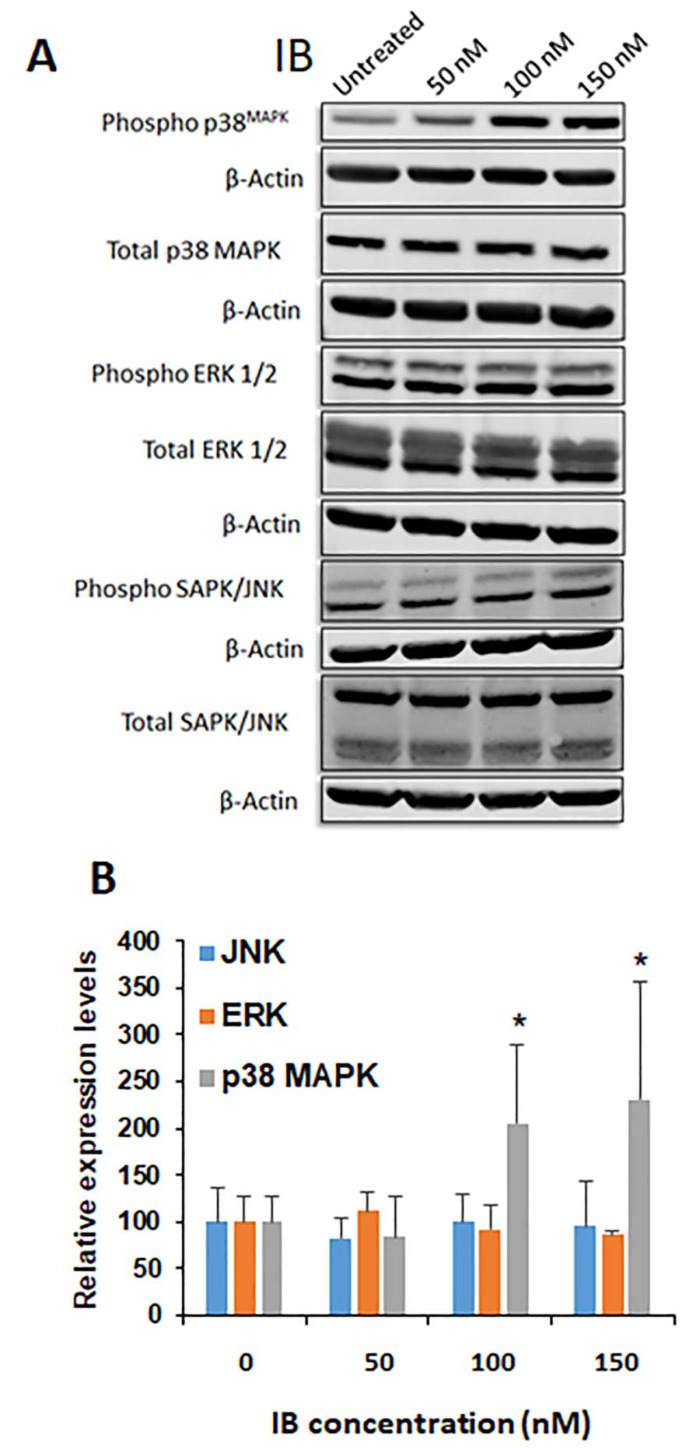
IB induces phosphorylation of p38 MAPK but not other MAPK family members. (**A**) MV4-11 cells were treated for 4 h with the indicated doses of IB. Western blot analyses were performed to compare phospho-signal to total signal for the following signaling pathway molecules: p38 MAPK, ERK1/2, SAPK/JNK. (**B**) Serial replicate MV4-11 cells treated the same as in panel A (4 h) were quantified and statistically analyzed to show the increase in p38 MAPK phosphorylation obtained at 50 nM, 100 nM, and 150 nM (*n* = 6). As controls, both ERK1/2 (*n* = 6) and SAPK/JNK (*n* = 3) were also tested following IB treatment. The untreated control cells were normalized to 100 but the average error was used for 2-tailed T-test statistical analysis. *p* values calculated relative to untreated control for p38 MAPK phosphorylation were significant as indicated. * *p* < 0.05.

Having ruled out UPR as the mechanism of action, we performed an additional screening of potential genes involved in ER–mitochondria interactions as related to calcium release and cell death. The list included Fis1, Ip3r, Mfn1, Drp1, Grp75, Mtorc2, Vdac1, and Bap31. The expression of those genes was examined by qRT-PCR and interestingly, Drp1 stood out as a candidate regulator that was significantly upregulated in Flt3-ITD^+^ vs. Flt3-ITD WT cells (Figure 6A). Therefore, we next focused on Drp1 protein levels by Western blot. Interestingly, while total Drp1 protein was only marginally increased at baseline in Flt3-ITD^+^ cells (Figure 6B,D), there was a much larger and more significant decrease in phosphorylated Drp1 in these cells (Figure 6C,E). Since, during oxidative stress, the activation of stress-activated protein kinases (SAPKs) such as p38MAPK leads to ROS-induced phosphorylation can cause cell cycle arrest, we also performed Western blot for p-p38, total p38, p-ERK1/2, total ERK1/2, p-SAPK/JNK, and total SAPK/JNK. Notably, only p-p38 MAPK levels were induced by IB treatment (Figure 7A,B).

### 3.6. Imipramine Blue and Pimozide Functions through Liberation of ROS and Quenching of ROS Eliminates Their Combined Effectiveness

**Figure 8:**
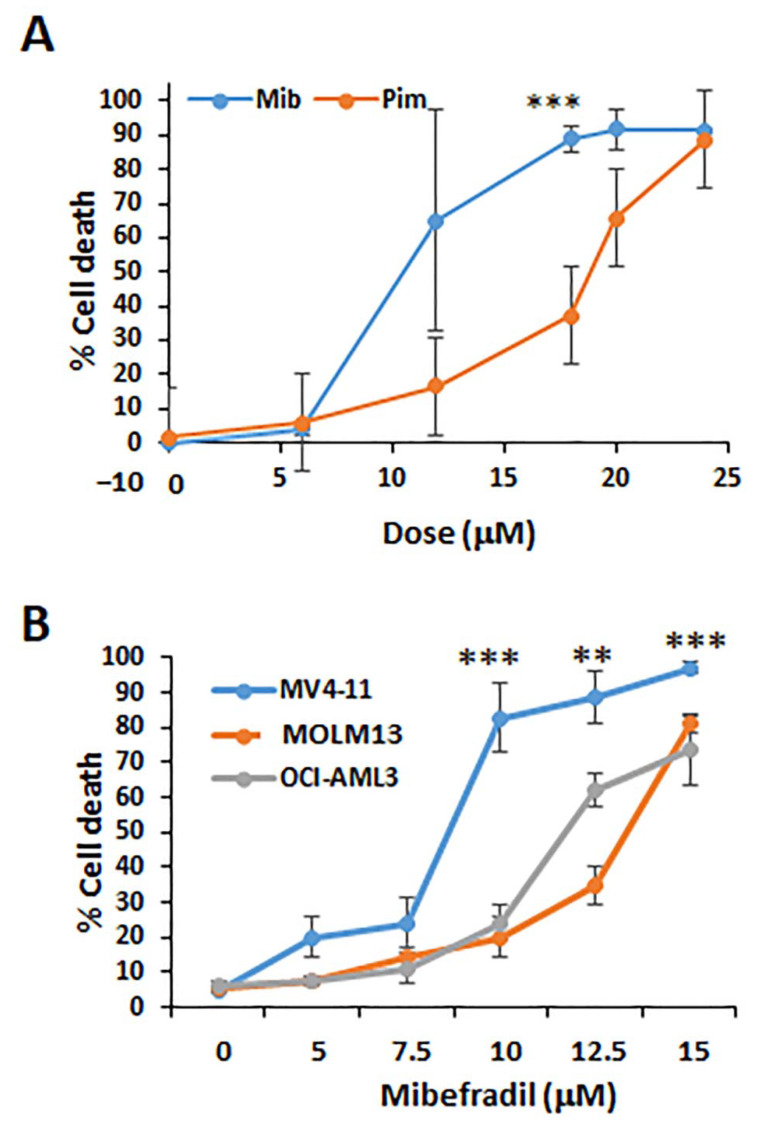
Pimozide and mibefradil have comparable activity on MV4-11 cells. (**A**) Pimozide and mibefradil were compared to determine whether their cytotoxicity profiles are similar in MV4-11 cells treated for 48 h with either drug. *p* values are relative to OCI-AML3 cells for *n* = 3. (**B**) Mibefradil was also tested in MV4-11 cells for 48 h and compared with MOLM13 and OCI-AML3 cells that express either low levels of Flt3-ITD or no Flt3-ITD, respectively. *p* values are relative to OCI-AML3 cells. ** *p* < 0.01; *** *p* < 0.001.

Having defined new mechanisms of action for both IB and pimozide which did not appear to involve a direct role for hydrogen peroxide, we next wanted to revisit IB/Pim and try to define where they converge. The hypothesis to be tested was that superoxide is the key target of relevance. Since pimozide is a known calcium channel blocker, we also wanted to determine whether other agents with the same calcium channel blocking activity were similarly effective at killing MV4-11 cells. First, we demonstrated that pimozide is effective at inducing cell death in MV4-11 cells with a dose–response curve that was comparable but about two-fold different from that of Mibefradil at the IC_50_ concentration (Figure 8A). Mibefradil was also more specific for MV4-11 cells than for MOLM13 or OCI-AML3 cells (Figure 8B).

**Figure 9:**
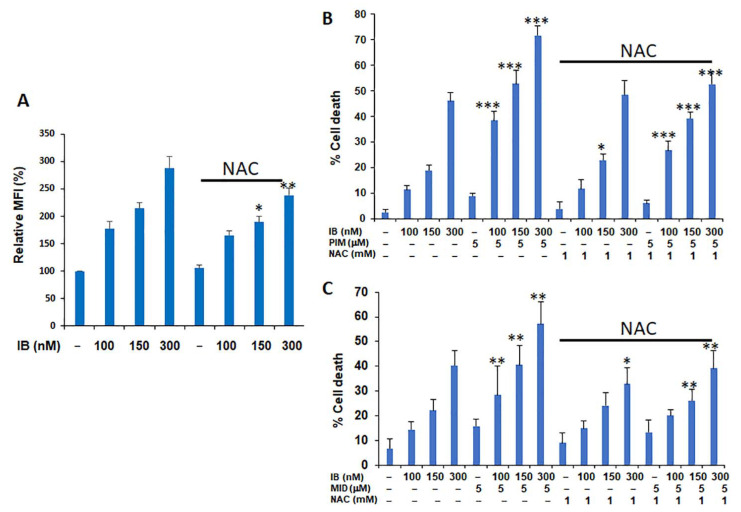
N-acetylcysteine effectively reverses IB-mediated cell death alone or in combination with pimozide or mibefradil. (**A**) MitoSox levels were assessed following IB treatment and potential reversibility was determined with NAC treatment (1 mM) for 4 h. Comparison of IB + PIM (Panel (**B**); *n* = 5) and IB + Mib (Panel (**C**); *n* = 6) on MV4-11 cells for 48 h was performed. Treatment of N-acetylcysteine (NAC) was also performed to test for reversal of cytotoxicity. Comparisons on the left were comparing IB vs. Pim or IB vs. Mib; *p* values on the right under the NAC heading compared +/- NAC for the same combination doses of IB/Pim or IB/Mib. * *p* < 0.05; ** *p* < 0.01; *** *p* < 0.001.

To test whether increased superoxide levels following IB treatment could be reversed with N-acetylcysteine (NAC), MV4-11 cells were treated with or without NAC treatment (Figure 9A). NAC as able to significantly reverse IB-mediated cell death at the 150 nM and 300 nM concentrations. More importantly, NAC treatment was equally effective at the reversal of either IB/Pim (Figure 9B) or IB/Mib (Figure 9C) combination treatments. Therefore, these data demonstrate a unique ROS-mediated mechanism for combinatorial interaction of IB with calcium channel inhibition. Notably, this therapy is a novel phospho-STAT5-independent approach for targeting Flt3-ITD^+^ AML cells.

## 4. Discussion

Acute myeloid leukemia is characterized by poor prognosis and a high relapse rate despite initial remission. Since lack of durable remission is a clinical problem, detection of minimal residual disease in AML patients is an important clinical approach to early detection of relapsing clones. Multi-kinase (e.g., sorafenib) and more selective class III receptor tyrosine kinase inhibitors (TKIs) (e.g., quizartinib) are being actively investigated in clinical studies. Despite the recent flow of new agents entering trials, TKI monotherapy has not been a magic bullet. Currently targeting Flt3-ITD expressing populations and development of newer generation TKIs (e.g., gilteritinib) has been a major objective. The approval of tyrosine kinase inhibitors has been limited to their use as single agents. In contrast, TKIs combined with chemotherapy or following hematopoietic stem cell transplantation [31] offer the potential to reduce side effects. Of these approaches, combination treatments based on the BH3 mimetic have proven to be the most effective clinically [22,32,33,34]. MLL-AF9 is co-expressed in the two Flt3-ITD^+^ cell lines used in this study and has been effectively targeted by combinations of conventional chemotherapy drugs and BH3 mimetics [35]. This mutation is known to enhance Bcl-2/Mcl1 expression, which can be synergistically targeted. Studies that further optimize and improve upon the early success of BH3 mimetics have significant potential. Recently, combinations of BH3 mimetics targeting Bcl-2 and Mcl1 were effective in AML mouse models in vivo [36].

In this study, double and triple combinations based on a BH3 mimetic backbone are shown to overcome some of the mechanisms of survival in the face of Bcl-2/Bcl-X_L_ inhibition alone. Although Bcl-X_L_ inhibition is obviously avoided in patients to bypass thrombocytopenia, we used early-generation and later-generation drugs in this study and found that for in vitro mechanistic and pre-clinical evaluation, they were functionally equivalent. We recently showed that imipramine blue and pimozide are highly synergistic in Flt3-ITD^+^ AML cells [21]. Therefore, potent and selective cytotoxic effects alone and in combination with pimozide as the “synergizer” backbone for Flt3-ITD^+^ AML may also be promising for clinical development. Imipramine blue induces calcium release from the ER/lysosomes and can inhibit tyrosine phosphorylation of STAT5. The calcium/reactive oxygen species/STAT5 signaling axis is a potential therapeutic target for high risk AML [37]. There are important implications regarding the underlying mechanism whereby proliferation might be targeted by targeted oxidative phosphorylation. Notably, Figure 3 demonstrated that K562 cells were not sensitive to the double and triple drug combinations. Since K562 cells are CML-like BCR-ABL^+^ cells, they have increased STAT5/mTOR activation, yet they are metabolically wired differently from AML cells. Therefore, targeting activated tyrosine kinase driven STAT5 is not perfectly straight-forward. There are other factors that need to be considered in regard to drug therapy and the induction of apoptosis. Further work in this area is needed to improve upon TKI treatments.

Activated Akt leads to increased amino acid and glucose uptake to support rapidly dividing cells. Synergistic combination approaches have been tested in a variety of cell types, including leukemia cells. Although rapalogs have been used for many years, they have not been replaced by newer mTORC1/2 inhibitors in the clinic. The triple combinations were the most effective and thus warranted further development in patients not characterized by homozygous Flt3 mutations. Instead, they were best suited for intermediate/low risk AML patients and thus provide a broader range of options. Multi-drug combinations in chemotherapy have been standard of care for many decades. Therefore, combining targeted agents is also a rational approach that is gaining acceptance, especially as wider understanding of the genetic make-up of individual patient is available to guide clinical decision-making. By leveraging substantial interaction among these agents, there are now therapeutic opportunities. Phase I and Phase II clinical studies are ongoing with BH3 mimetics, which already show promise in IDH-mutated relapsed refractory AML patients (American Society of Hematology meeting, unpublished). Profiling of oxidative phosphorylation and associated metabolic rewiring may lead to discrete combinations with improved efficacy to be used in a patient-by-patient case. Furthermore, beyond our in vitro studies shown here, targeting STAT5 activation with pimozide promises to also target leukemia stem cell self-renewal in vivo, which might further reduce the relapse rate.

Surprisingly, in this study, we found that imipramine blue and pimozide synergize potently through mechanisms that have not been described previously for these drugs in AML. This phospho-STAT5-independent Drp1/p38/ROS signaling cascade is engaged through IB. Although we showed that ROS are ultimately responsible, we did not use pharmacologic targeting of Drp1 or p38 MAPK because of potential side effects that would make interpretation difficult. The combination therapy and is quite effective at low nM concentrations. Notably, the gentian violet moiety of IB has previously been shown to target mitochondrial apoptosis [38] and it may be the more active moiety of IB in Flt3-ITD^+^ AML cells, as supported by Figure 4B. Further structure–function studies of IB may be warranted in the future. It is relevant to point out that this molecular pathway and mitochondrial membrane integrity in general has been described as a potential source of “pre-relapse” clones during leukemia evolution [39]. Therefore, innovative drug combinations that target the relapse-fated clones would be very clinically useful.

## 5. Conclusions

Drug combinations are a very important approach in modern cancer biology and finding the best combinations for high-risk diseases such as AML is a high priority. We approached new ways to exploit mitochondrial ROS sensitivity in AML cells by using several agents targeting either the BH3-domain containing proteins, mTOR, or calcium regulation. While seemingly disparate, all of these agents converge on mitochondrial function (see Graphical Abstract), and we have shown dependence on MitoSox for their combined actions. In summary, the agents described here provide proof-of-principle for combinatorial treatments that effectively overcome resistance and induces selective mitochondrial cell death in AML cells independent of previously described inhibition of phospho-STAT5 by imipramine blue and pimozide. Tailored treatment regimens based on two- or three-drug combinations suggest that precision medicine for high-risk AML might be achieved in the absence of traditional chemo-radiotherapy.

## Data Availability

For primary research materials or data please direct all correspondence to Bunting (kevin.bunting@emory.edu).

## Supplementary Materials

The following are available online at https://www.mdpi.com/article/10.3390/antiox10060956/s1, Figure S1: Comparison of pimozide combinations with the ROS modulators, rotenone, metformin, and DPI, Figure S2: ABT-263 and ABT-199 have equivalent synergistic cytotoxicity with mTOR inhibition in AML cells.
