# Supplementary material for: Pimozide and Imipramine Blue Exploit Mitochondrial Vulnerabilities and Reactive Oxygen Species to Cooperatively Target High Risk Acute Myeloid Leukemia
Source: Antioxidants (Basel). 2021 Jun 15;10(6):956. doi: 10.3390/antiox10060956 (PMC8232307; doi:10.3390/antiox10060956)
Supplement: Supplementary file 1 [file antioxidants-10-00956-s001.zip › antioxidants-1240104-supplementary.pdf]

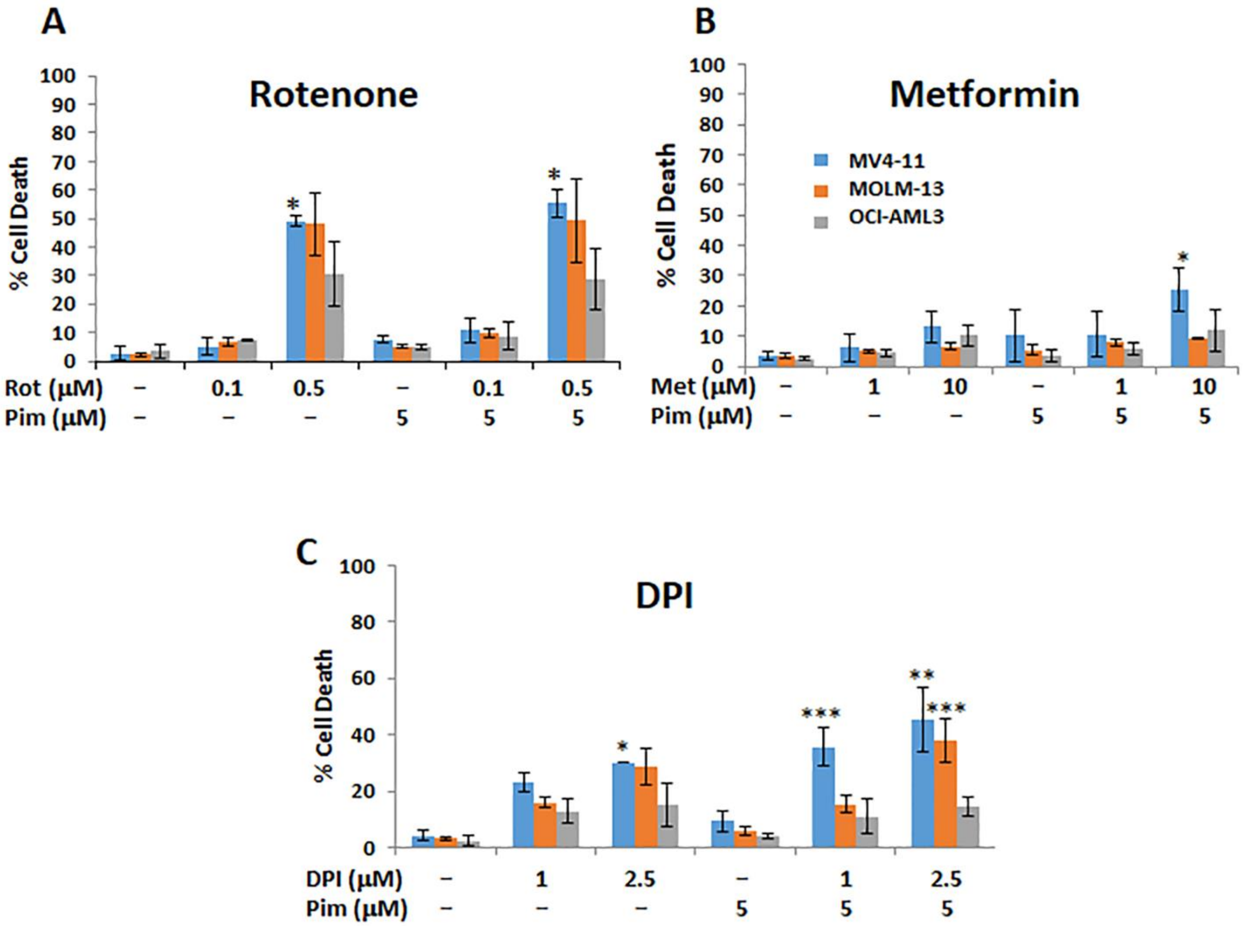

Figure S1. Comparison of pimozide combinations with the ROS modulators, rotenone, metformin, and DPI. Cells were treated for 48 hours with rotenone (Rot), metformin (Met), or diphenyleneiodonium (DPI) and the percentage of cell death was determined by trypan blue exclusion assay. N=3 for all three drugs tested on MV4-11, MOLM-13, and OCI-AML3 cells. P values were calculated relative to OCI-AML3 at the highest concentration of Rot (Panel A), Met (Panel B), or DPI (Panel C). *P<0.05; **P<0.01; ***P<0.001.

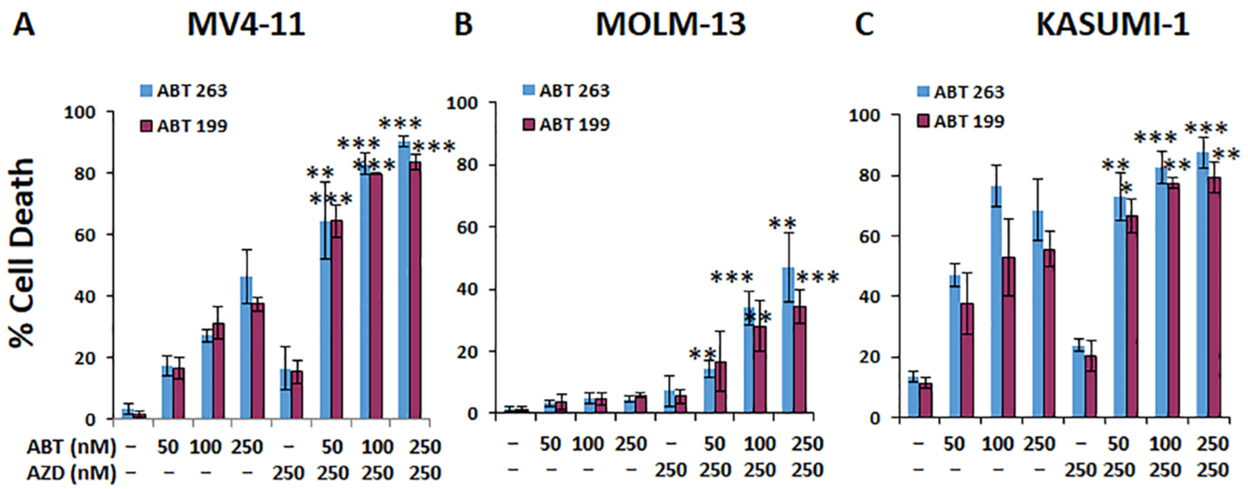

Figure S2. ABT-263 and ABT-199 have equivalent synergistic cytotoxicity with mTOR inhibition in AML cells. Three cell lines were examined for sensitivity to treatment with ABT-263 and AZD 8055 for 48 hours at the indicated drug concentrations. These cell lines included: MV4-11 (**panel A**, high responder; homozygous Flt3-ITD; N=3) and MOLM-13 cells (**panel B**, intermediate responder; heterozygous Flt3-ITD; N=4) and Kasumi control cells (**panel C**, non-responder; Flt3 wild-type; N=3). P values were calculated relative to ABT 50 (nM) group. *P<0.05; **P<0.01; ***P<0.001.
